# Weather conditions and legionellosis: a nationwide case-crossover study among Medicare recipients

**DOI:** 10.1017/S0950268824000979

**Published:** 2024-10-17

**Authors:** Timothy J. Wade, Carly Herbert

**Affiliations:** 1 United States Environmental Protection Agency, Office of Research and Development, Research Triangle Park, NC, USA; 2 Oak Ridge Associated Universities, United States Environmental Protection Agency, Office of Research and Development, Research Triangle Park, NC, USA

**Keywords:** Legionella, Legionellosis, weather, case-crossover, medical records, precipitation, humidity, temperature

## Abstract

Legionellosis is a respiratory infection caused by *Legionella* sp. that is found in water and soil. Infection may cause pneumonia (Legionnaires’ Disease) and a milder form (Pontiac Fever). *Legionella* colonizes water systems and results in exposure by inhalation of aerosolized bacteria. The incubation period ranges from 2 to 14 days. Precipitation and humidity may be associated with increased risk. We used Medicare records from 1999 to 2020 to identify hospitalizations for legionellosis. Precipitation, temperature, and relative humidity were obtained from the PRISM Climate Group for the zip code of residence. We used a time-stratified bi-directional case-crossover design with lags of 20 days. Data were analyzed using conditional logistic regression and distributed lag non-linear models. A total of 37 883 hospitalizations were identified. Precipitation and relative humidity at lags 8 through 13 days were associated with an increased risk of legionellosis. The strongest association was precipitation at day 10 lag (OR = 1.08, 95% CI = 1.05–1.11 per 1 cm). Over 20 days, 3 cm of precipitation increased the odds of legionellosis over four times. The association was strongest in the Northeast and Midwest and during summer and fall. Precipitation and humidity were associated with hospitalization among Medicare recipients for legionellosis at lags consistent with the incubation period for infection.

## Introduction

Legionellosis is a respiratory infection caused by *Legionella* sp. bacteria that occur naturally in water and soil. Infection may cause severe pneumonia (Legionnaires’ Disease) or a milder, flu-like illness (Pontiac Fever), collectively known as Legionellosis [1]. In the United States, most cases (>90%) are caused by *Legionella pneumophila.* Legionnaires’ Disease is especially dangerous for those 50 years or older, who have weakened immune systems, or chronic lung conditions [2]. Over 10 000 are hospitalized annually with Legionnaires’ Disease in the United States [3] and the case-fatality rate is approximately 10% [4]. The incubation period generally ranges from 2 to 14 days [4]. Pontiac fever is rarely reported, making up less than 1% of known legionellosis cases [5].

*Legionella* sp. is found naturally in fresh water where they survive as intracellular parasites of free-living protozoa [1]. They colonize biofilms in water systems such as showerheads, cooling towers, hot tubs, fountains, hot water tanks and plumbing systems [2], and grow well in warm water (25–45°C) [6]. Factors influencing the growth of *Legionella* in water systems include inadequate disinfectant and failure to maintain water temperatures outside of those that favour growth [7]. The primary route of exposure is inhalation of aerosolized bacteria [4] and infections have been associated with sources from distances over 3 km [6].

Outbreaks are common in hotels, workplaces, senior living facilities, hospitals, and cruise ships [7], but most cases are sporadic [2] and outbreaks only represent about 4% of cases [1]. Recently, Legionnaires’ Disease has risen in the United States, Europe, and elsewhere [8]. The reason is not known, but may be due to increased detection, ageing water systems, and an ageing population.

Changes in weather patterns may also explain the rise in cases. Legionnaires’ Disease peaks in the late summer and early fall, suggesting weather may affect transmission [9]. Warm temperatures and wet, humid conditions may support the proliferation and survival of *Legionella* sp. in water systems and the environment [10].

In 2005, a case-crossover study in the Philadelphia area found precipitation and humidity in the 6–10 days prior were associated with legionellosis [11]. Similar studies have been conducted in different areas including Belgium [12], the Netherlands [10], the United Kingdom [13, 14], Taiwan [15], Spain [16], Switzerland [17, 18], New Zealand [19], Korea [20], Japan [21], the United States [9, 22, 23] and several European countries [24]. A recent review concluded: ‘that increased precipitation, temperature and relative humidity were positively associated with the incidence of LD’. [25].

Although the topic has been rather extensively studied, results vary by location and study design. Whereas most studies found positive associations with precipitation, humidity or temperature, the important exposure lags varied widely, from 1 day to 20 weeks. Many of the studies cannot be generalized as they had relatively few cases (i.e., fewer than 1000 cases) [10, 11, 19, 23]; took place in a restricted area [10, 11, 13–15, 18–21, 23]; or a relatively short time period (<5 years) [14, 18, 19, 26]. Several studies could not address specific exposure lags as they aggregated exposures and cases by week or month, or considered only broad geographic aggregation of cases (e.g., by state) [9, 17, 22, 24, 26, 27].

We extend the previous work to a large sample of legionellosis cases using records from the Centers for Medicare and Medicaid Services (CMS). These files include records of hospital visits for all Medicare beneficiaries which covers over 95% of the US population over 65 years of age. The objectives were to explore associations between weather and legionellosis cases, specifically the role of temperature, precipitation and relative humidity, including extreme days. We compared geographic and seasonal differences, interactions among the exposures and changes in the associations over time. We explored non-linear associations and distributed lags using distributed lag non-linear models [28].

## Methods

### Case data

The CMS MedPAR database contains information on hospital and nursing facility visitations that were covered by Medicare. It includes up to 25 International Classification of Disease (ICD) Diagnostic Codes version 9 or version 10 (ICD-9 and ICD-10) for each visit. ICD-10 codes were used starting in 2015 and included a diagnostic code for both Legionnaires’ Disease and Pontiac Fever (A481 and A482) while ICD-9 codes only included a code for Legionnaires’ Disease (42824).

We extracted records with any ICD codes for Legionnaires’ Disease and/or Pontiac Fever for the years 1999–2020. From each record, we obtained demographic information, residential zip code, and admission and discharge dates. If there were multiple visits only the first visitation, as indicated by beneficiary identification number, was retained.

### Weather data

Daily weather (total precipitation, minimum, mean and maximum temperature, mean dew point temperature, minimum and maximum vapour pressure) was obtained from the PRISM Climate Group (https://prism.oregonstate.edu). The R library ggmap [29] was used to geocode and return the latitudes and longitudes from the centre of the zip code of the case residence and then used to query the PRISM database. Weather data were obtained for each location ±30 days from the admission or control date. Relative humidity was estimated according to the following formula [30]:

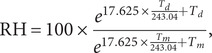

where *T_d_* is the daily mean dewpoint temperature in Celsius and *T_m_* is the daily mean temperature in Celsius. Because only daily mean dew points were available in the PRISM data, the relative humidity represents a daily average value.

Temperature, precipitation, and relative humidity were considered on a continuous scale and classified into three binary definitions of high or threshold days shown in Table 1. PRISM weather data were not available outside the continental US so cases from these areas were excluded from the study.

**Table 1.** Weather summaries for 0–20 days lag for cases and controls

	*N*	Median	Mean	25th/75th	Min/Max
Precipitation (all days) (cm)	1 783 782	0.00	0.34	0.00/0.20	0.000/44.17
Precipitation (days with precipitation) (cm)	676 126	0.41	0.89	0.13/1.14	0.02/44.17
Relative humidity (%)	1 783 782	69.74	68.57	60.06/78.50	4.17/100
Temperature, daily maximum (°C)	1 783 782	23.6	20.9	14.6/28.6	−29.0/48.7
High Days – Definition 1					
Daily Precipitation >0 cm	676 126 (37.9%)	0.41	0.89	0.13/1.14	0.03/44.17
Relative Humidity >80% (daily average)	385 153 (21.6%)	86.3	87.48	82.73/91.31	80.00/100
Temperature > 29°C (daily maximum)	413 196 (23.16%)	31.5	31.9	30.2/33.1	29.0/48.7
High Days – Definition 2					
Daily Precipitation > 1.27 cm (0.5 inches)	150 578 (8.4%)	2.13	2.66	1.63/3.07	1.30/44.17
Relative Humidity >90% (daily average)	117.816 (6.6%)	94.08	94.62	91.83/97.26	90.00/100
Temperature >32°C (daily maximum)	167 080 (9.4%)	33.5	34.1	32.7/34.8	32.1/48.7
High Days–Definition 3					
Daily Precipitation >2.54 cm (1 inch)	54 856 (3.1%)	3.53	4.17	2.95/4.65	2.57/44.17
Relative Humidity >95%(daily average)	48 860 (2.74%)	97.99	97.94	96.32/100	95.00/100
Temperature >35°C (daily maximum)	38 459 (2.16%)	36.6	37.3	35.7/38.3	35.1/48.7

### Statistical analysis

We used a time-stratified bi-directional case-crossover design which has been widely used to study transient exposures and acute health effects [31], including previous studies of legionellosis and weather conditions [11, 12, 14, 18]. In this design, time is stratified *a priori*, and within these strata, cases are defined as the date of the event (hospital admission) and control periods are defined as dates when the event did not occur. Exposure histories are compared for days when the event was experienced (case) to days when not experienced (controls). The time-stratified bi-directional approach has been demonstrated to reduce bias resulting from trends in the exposure series compared to other approaches such as uni-directional selection of only controls that precede the event and bi-directional control selection at fixed intervals [32, 33]. This design controls for characteristics which do not vary meaningfully over the observation period (e.g., age, sex, race, location). Cases and controls were stratified by year and calendar month, and matched by day of week, effectively controlling for seasonal effects and time trends [33, 34]. To account for *Legionella* incubation, cases and controls were separated by 14 days. Therefore, depending on when the case occurred in the month, there could be one or two controls, occurring before or after the case.

Conditional logistic regression was used to model the outcome (case/control) as a function of exposure variables. Lags for precipitation, temperature and relative humidity of up to 20 days were included. Correlations among lagged predictor variables were examined using Pearson correlation. Multicollinearity was evaluated using generalized linear models with binomial distributed errors to estimate variance inflation factors (VIF). Variables with VIFs greater than 5 were transformed to reduce collinearity. Temperature, precipitation and relative humidity were also modelled separately (single exposure models).

Separate models were fit by season: Winter (December, January and February); Spring (March, April, May); Summer (June, July, August); and Fall (September, October, November). Models were also stratified by geographic region (West, Midwest, Southeast, and Northeast) as defined by the National Geographic Society (https://education.nationalgeographic.org/resource/united-states-regions/); and five-year time-periods.

A model with two-way interaction terms at the same lag was fit (i.e., interaction terms for temperature and precipitation; precipitation and relative humidity; and relative humidity and temperature) as well as reduced models with interactions for selected lags, informed by the results of the primary models without interaction terms.

To reduce Type I errors from multiple comparisons and large sample size, *p*-values and confidence intervals for each model were adjusted using the Bonferroni correction.

Distributed lag non-linear models (DLNM) were used to explore non-linear responses and lag distributions using the R library dlnm [28]. In these models, a cross-basis is specified which provides the shape of both the lagged distribution and the response distribution. We considered natural cubic spline functions for temperature and humidity with knots at the 10th, 50th and 90th percentile. Because precipitation was highly skewed, knots at the 10th, 90th, and 99th percentiles were used to account for extreme precipitation. Natural cubic splines were used for the lag distribution with knots at 2, 7, and 14 days, corresponding to the boundaries and middle of the incubation period for *Legionella* infection. We also fit distributed lag models for high days using natural cubic splines with knots at 2, 7, and 14 days. For DLNMs, lags of 25 days were included to allow increased flexibility in the lag distribution.

All data analyses were conducted using R 4.2.3 and RStudio 2023.03.0. In addition to the dlnm and ggmap libraries mentioned above, the ggplot2 library was used to develop figures [35].

## Results

### Legionellosis cases

**Figure 1.**
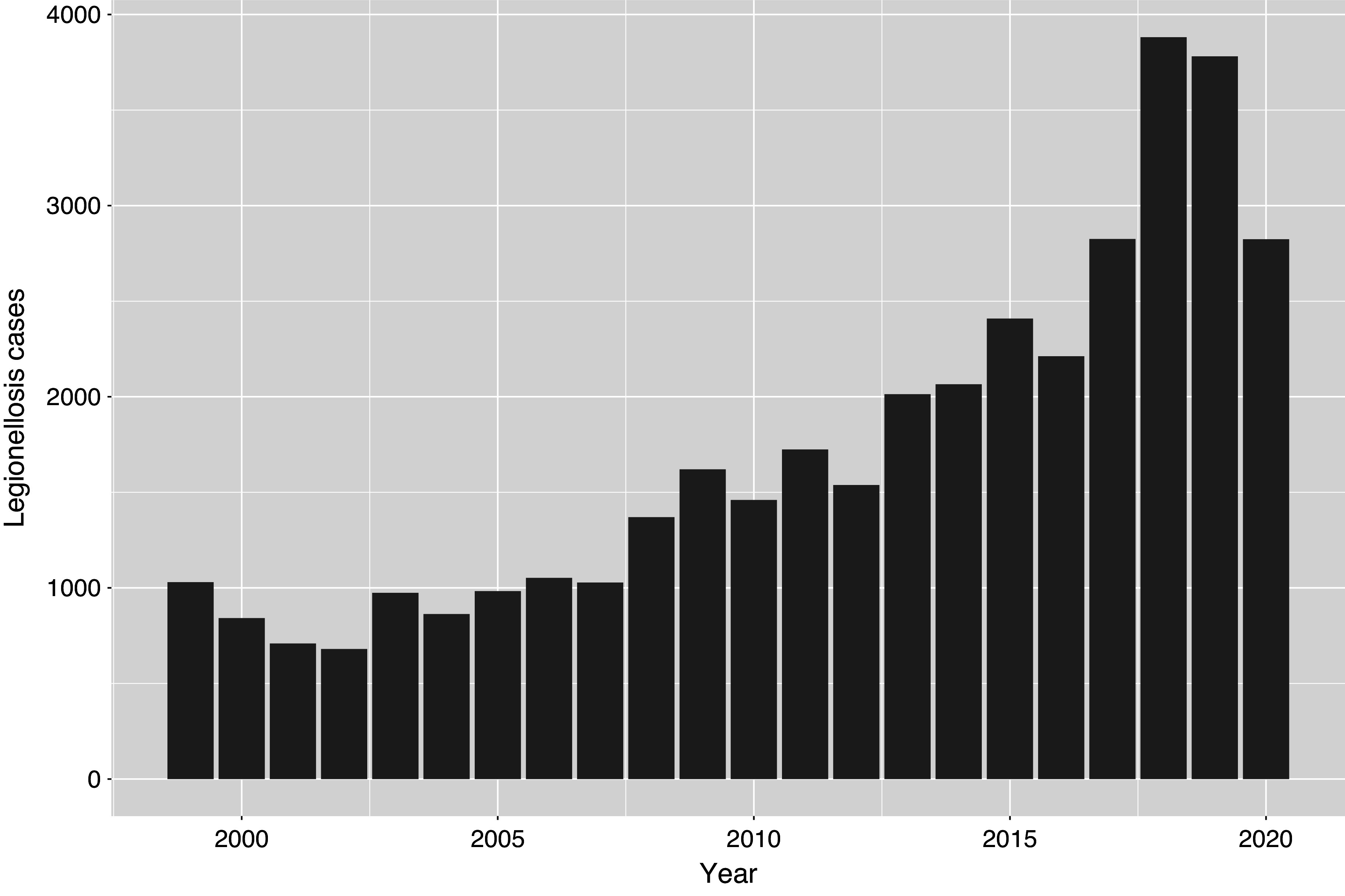
Legionellosis hospitalizations among Medicare recipients.

**Figure 2.**
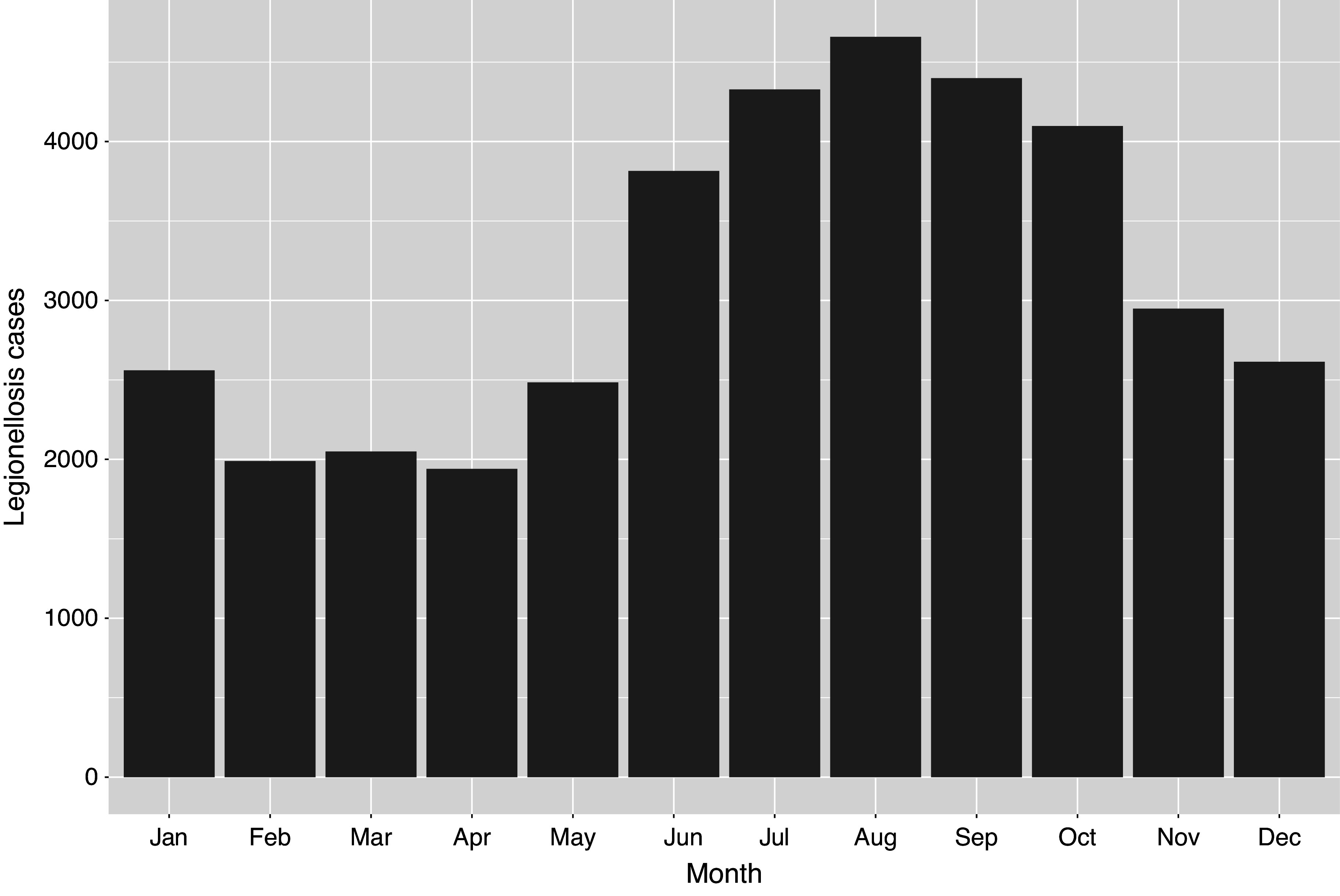
Legionellosis hospitalizations among the Medicare eligible population, by month.

Initially, 44 399 records with an ICD code for legionellosis were extracted. Following the removal of repeated hospitalizations of the same individual for legionellosis (*n* = 6 343) and cases without PRISM data (*n* = 173), 37 883 cases remained. Most (58%) were male, and the median age was 73 years (range 13–106 years). Total cases (unadjusted for population) rose over the study period, from 680 in 2002 to 3 881 in 2018 (Figure 1). Cases peaked in July–September (Figure 2), with most cases in August and fewest in late winter/spring (February–April). Cases were most frequent in the Northeast and Midwest. After adjusting for age and population (directly standardized to the US 2010 population), highest rates were in Ohio, Rhode Island and New York and lowest in New Mexico and Washington (Supplementary Figure S1).

Most cases were diagnosed with Legionnaires’ Disease (*n* = 37 787, 96%). Only 96 cases were diagnosed with Pontiac Fever alone and 16 cases had a diagnosis of both. Of the Legionnaires’ Disease cases, 43% were the primary ICD diagnosis, and 81% of were within the first three ICD codes (representing the primary, second, third and diagnostic codes).

For the 37 883 cases, there were 47 059 matched controls. Most cases were matched to one control (28 707) and 9 176 were matched to two controls.

### Weather conditions

Weather data for 0–20 lags from the case admission and control date are shown in Table 1.

Consecutive daily temperatures were highly correlated (average 1 lag-day *r* = 0.93), whereas day to day precipitation had low correlation (average 1 lag-day *r* = 0.17). Relative humidity on consecutive days was moderate-strongly correlated (average 1 lag-day *r* = 0.65), but this declined to 0.37 after 6 days and declined thereafter. Mean-centering of temperature reduced average consecutive day correlations to *r* = 0.59 and this declined to *r* = 0.049 after four days and to *r* = −0.03 after six days. Variance inflation factors (VIFs) for temperature (lags 0–20) were unacceptably high (~8–13). Following the mean centering of temperature, all VIFs were below 3. VIFs below 5 are considered to indicate low multicollinearity [36]. All models used mean-centred temperature to reduce multicollinearity.

### Case-crossover models

**Figure 3.**
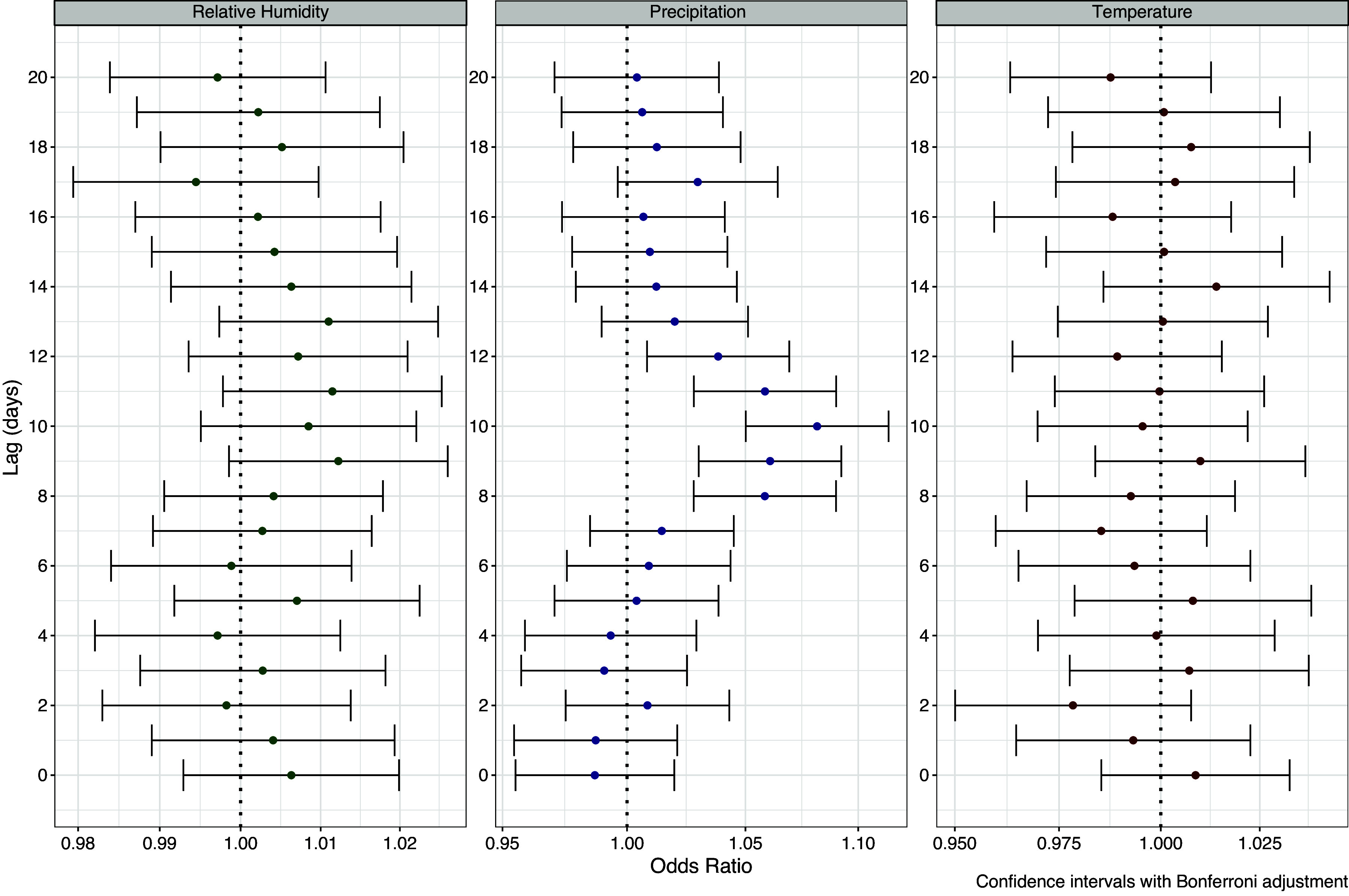
Associations between legionellosis cases and daily weather from case-crossover conditional logistic regression model. Odds ratios for relative humidity are for 5% increase; precipitation for a 1 cm increase, and temperature for 3°C increase.

Odds ratios were elevated for precipitation at lags 8–12 (Figure 3), peaking at lag day 10 (OR = 1.08, 95% CI = 1.05–1.11 per 1 cm). Odds ratios for relative humidity were elevated at lag days 9–13 (Figure 3), peaking at lag day 9 (OR = 1.01, 95% CI 0.99–1.03 per 5% increase) but were not significant following Bonferroni correction. Maximum daily temperature was not associated with Legionellosis (Figure 3). For single exposure precipitation models (Supplementary Figure S2) odds ratios were slightly higher (OR = 1.10, 95% CI 1.07–1.13 per 1 cm increase at lag day 10) and significant associations were also observed for lag days 13 and 17. For single exposure relative humidity models (Supplementary Figure S2), significant associations were observed for lag days 8–13, peaking at lag day 10 (OR = 1.02, 95% CI 1.01–1.03 for a 5% increase). Single-exposure temperature models showed no associations (Supplementary Figure S2).

**Figure 4.**
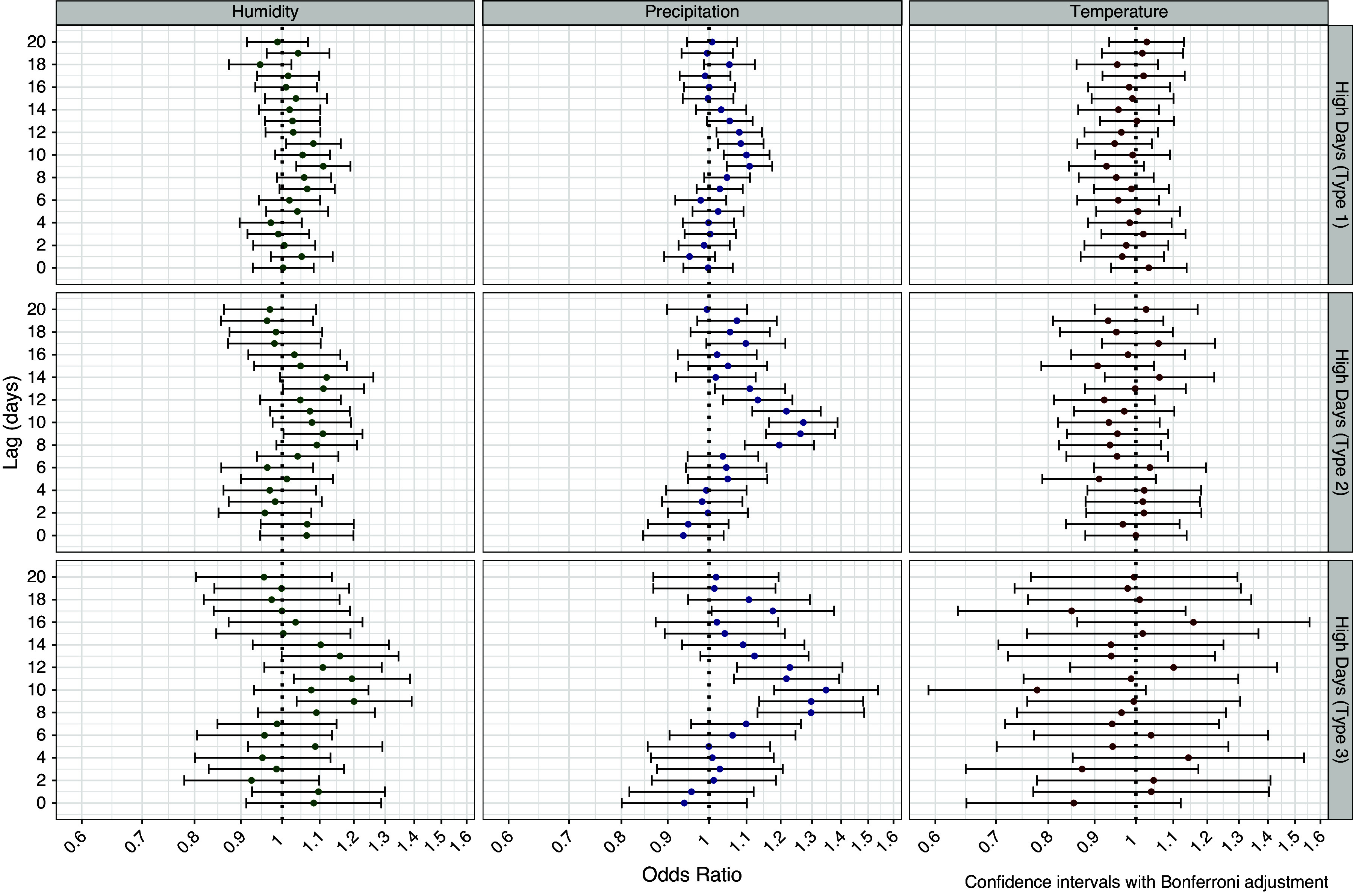
Associations between legionellosis cases and threshold effects of high weather days from conditional logistic regression case-crossover model. Type 1: any precipitation; relative humidity >80% and maximum daily temperature >29°C; Type 2: daily precipitation >1.27 cm (0.5 inch); relative humidity > 90% and maximum daily temperature > 32°C; and Type 3: precipitation >2.54 cm (1 cm); relative humidity > 95% and maximum daily temperature > 35°C.

Threshold models of days of high precipitation mirrored the associations of the continuous variables, with significantly elevated odds ratios at lags 8–12 and at lag day 17 for precipitation greater than 2.54 cm or 1 inch (Figure 4). At lag day 10 the odds of legionellosis hospitalization were 35% higher (OR = 1.35, 95% CI 1.18–1.54) on days with more than 2.54 cm (1 inch) of precipitation. Days of relative humidity above 90% on lag day 9 were associated with a 20% (95% CI 1.04–1.38) increased odds of legionellosis. High daily maximum temperatures were not associated with legionellosis. For single exposure high day models (Supplementary Figure S3), the associations with relative humidity strengthened with significant associations for lag days 7–12. In single exposure temperature models, significant negative associations were seen for lag days 8 and 9 when temperature exceeded 29°C, but this may have been confounded by an inverse association between precipitation and temperature. When precipitation variables for lags 8–12 were included, inverse associations with temperature were no longer evident.

### Region

**Figure 5.**
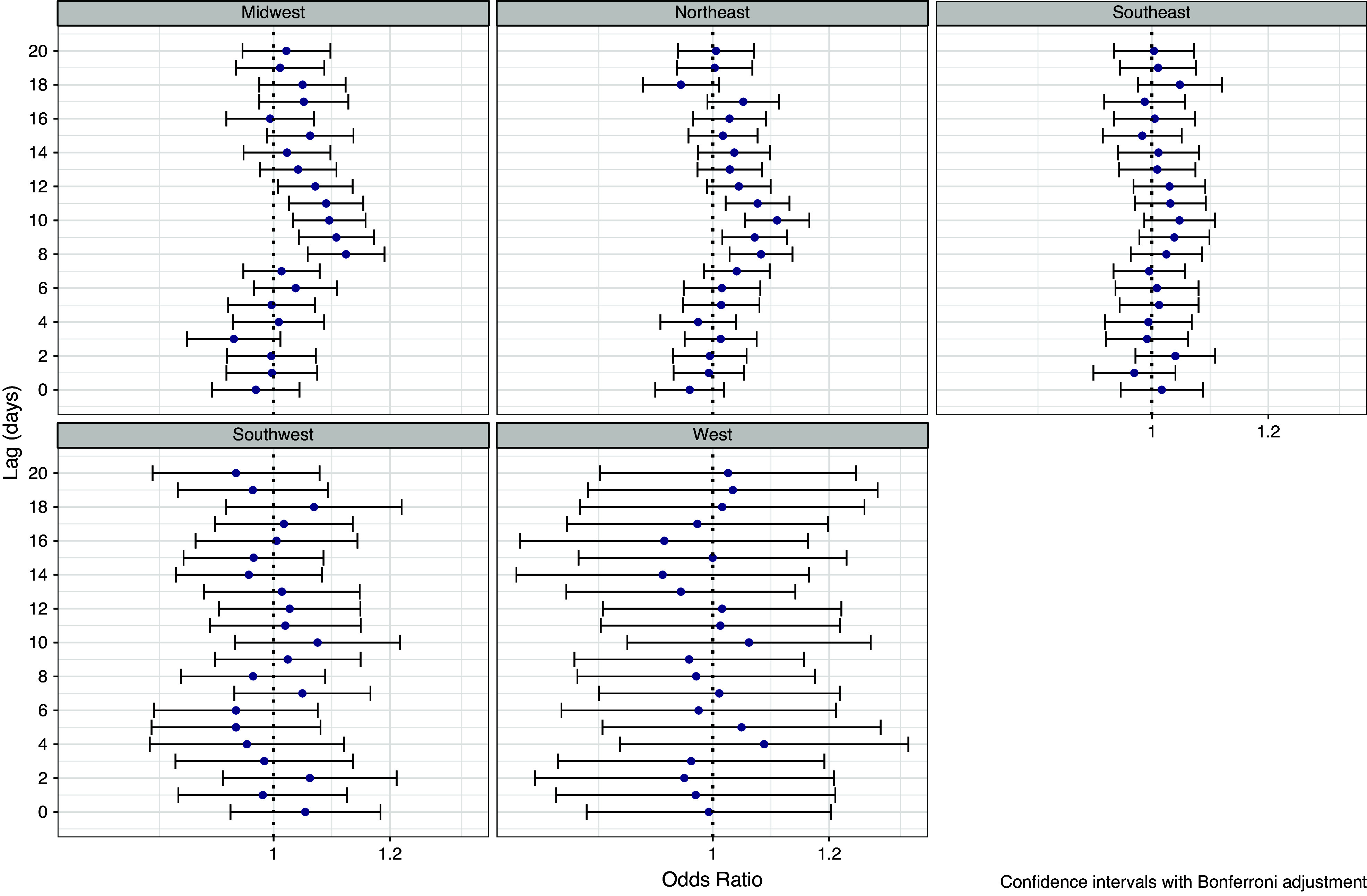
Associations between legionellosis cases and precipitation (odds ratios per 1 cm increase) from conditional logistic regression case-crossover model, by region.

**Figure 6.**
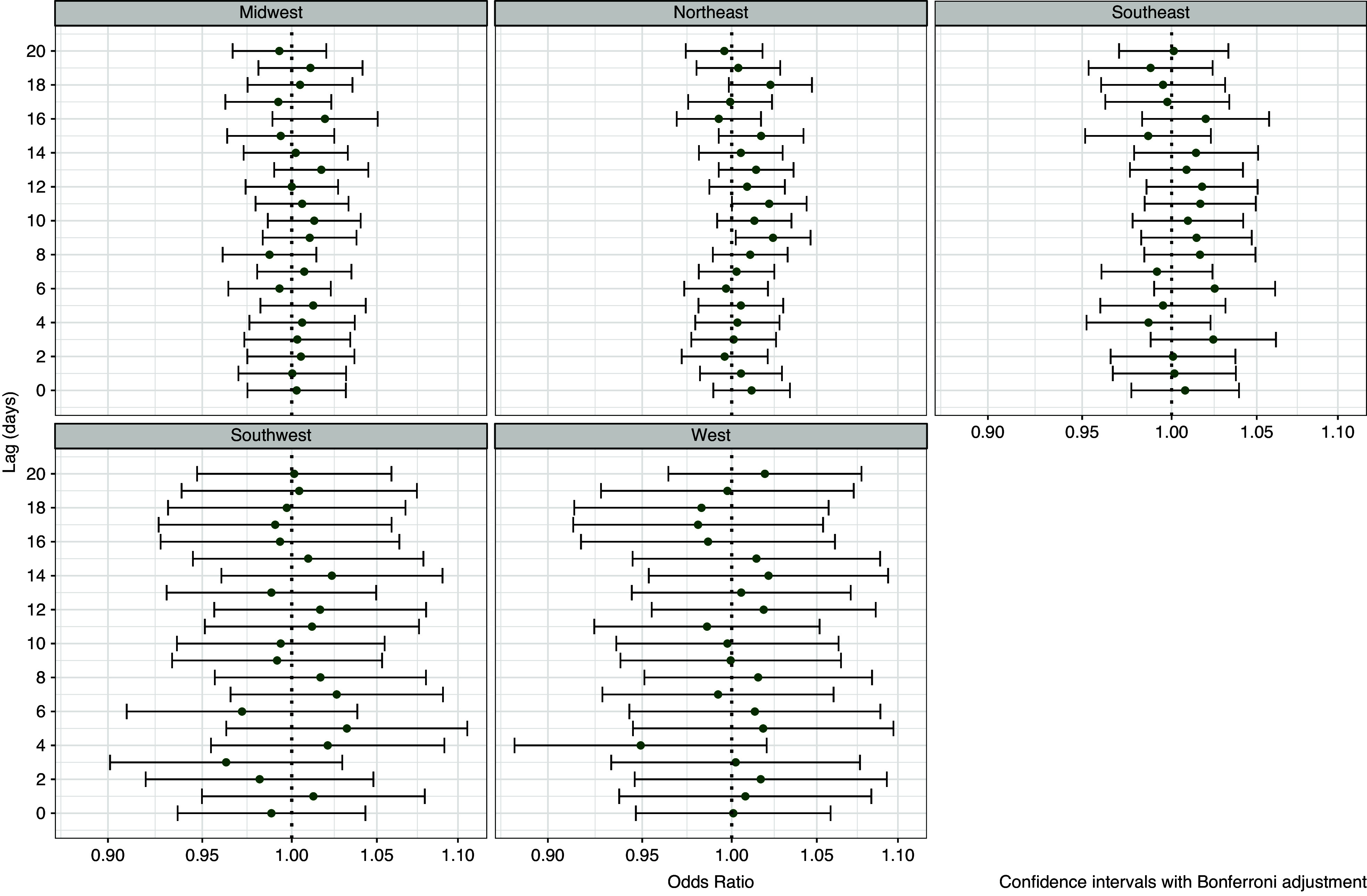
Associations between legionellosis cases and relative humidity (odds ratios per 5% increase) from conditional logistic regression case-crossover model, by region.

Associations between precipitation and humidity and legionellosis were strongest in the Northeast and the Midwest but generally absent in the West and Southwest (Figures 5 and 6 and Supplementary Figure S4). In the Northeast, significant associations were observed for precipitation at lag days 8–11 (peaking at lag 10, Figure 5) and for relative humidity at lag days 9 and 11 (Figure 6). In the Midwest, significant associations were also observed for precipitation at lag days 8–12 (peaking at lag 8, Figure 5) but no associations were observed with relative humidity (Figure 6). No associations were observed in the West and Southwest (Figures 5 and 6 and Supplementary Figure S4). In the Southeast, associations with precipitation (Figure 5) and relative humidity (Figure 6) were not significant, although patterns were consistent with the overall associations with elevated risks at lags 8–12. Models of high days showed similar patterns to the continuous models. In the Northeast, days with precipitation greater than 2.54 cm (1 inch) at lag day 10 (Supplementary Figure S5), were associated with a 50% increase in the odds of legionellosis (OR = 1.54, 95% CI = 1.24–1.90). In the Midwest, at lag day 8, precipitation of greater than 2.54 cm (1 inch) was associated with a 48% increase in legionellosis (Supplementary Figure S5, OR = 1.48, 95% CI = 1.13–1.88).

### Season

Precipitation was associated with legionellosis during the Fall and Summer at lag days 8–11 (Supplementary Figure S6). During Spring there was an association at lag day 10, and there were no associations during the winter months. The only association with relative humidity was during the Summer at lag day 10. In the Fall there were positive but non-significant associations with relative humidity at lags 9 and 11. There were no associations between legionellosis and temperature. Temperature was not included for high days due to very few high-temperature days in Winter (Supplementary Figure S7). Results reflected those in the continuous variable models, with strongest effects for precipitation and relative humidity in the Fall and Summer. At lag day 10, precipitation greater than 1.27 cm (0.5 inches) was also associated with legionellosis in the Spring and Winter (Supplementary Figure S7).

### Year

Effects were relatively consistent over the study period. Odds ratios for precipitation and relative humidity increased in 2011–2015 and 2016-2020 (Supplementary Figure S8). Strongest effects for each time-period were precipitation at lag day 10 (2000–2005; and 2016–2020), precipitation at lag day 8 (2006–2010) and precipitation at lag day 11 (2011–2015).

Models with two-way interaction terms showed no evidence of interaction. Reduced models including interaction terms for precipitation and humidity only for lag days 6–15 also did not indicate any significant interaction effects.

### Distributed lag non-linear models

Distributed lag non-linear model results are shown inFigures 7–12 and Supplementary Figures S9–S15. Odds ratios are relative to baseline values of zero precipitation, 60% relative humidity; and mean temperature.

**Figure 7.**
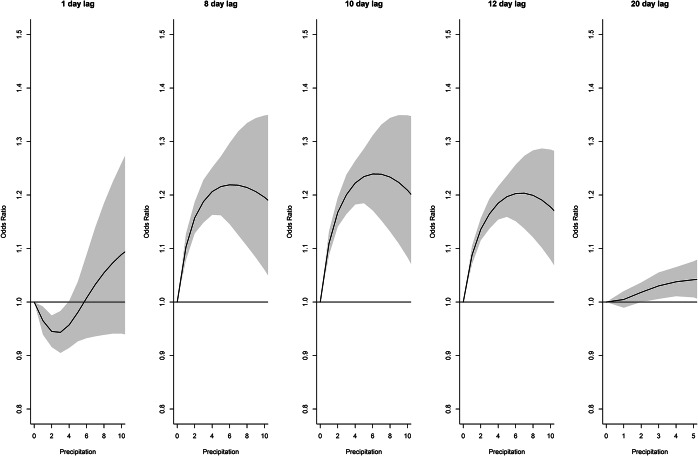
Distributed lag non-linear model for legionellosis cases. Effect of daily precipitation (cm) at selected lag days (odds ratios are relative to no precipitation).

**Figure 8.**
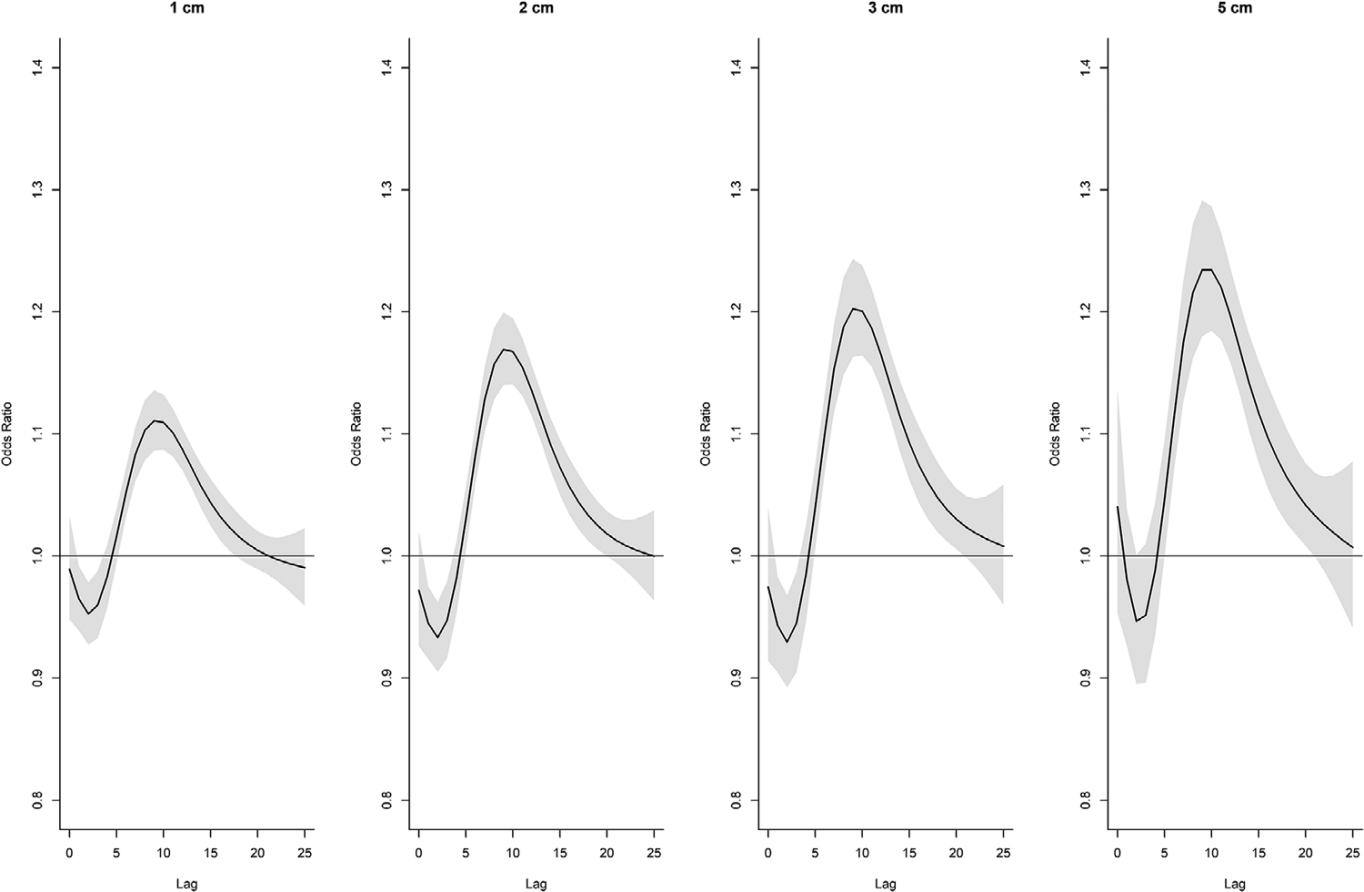
Distributed lag non-linear model for legionellosis cases. Effect of daily precipitation for selected totals over the lag period (odds ratios are relative to no precipitation).

For precipitation, the association at lag days 8, 10, and 12 (Figure 7) increased to about 7 cm and then declined above 7 cm. Days above 10 cm were excluded from the figures as estimates were extremely imprecise. The effect of precipitation over the lag period (Figure 8) was slightly decreased risk at lag days 1–2 followed by an increased risk at lags 6–20, peaking at lag day 10 and declining after lag day 20. The cumulative effect of 2 cm of precipitation over 20 days (Supplementary Figure S9) increased the odds of legionellosis 3.2 times (95% CI 2.38–4.36) and 3 cm of precipitation increased the odds of legionellosis 4.4 times (95% CI 2.94–6.60).

**Figure 9.**
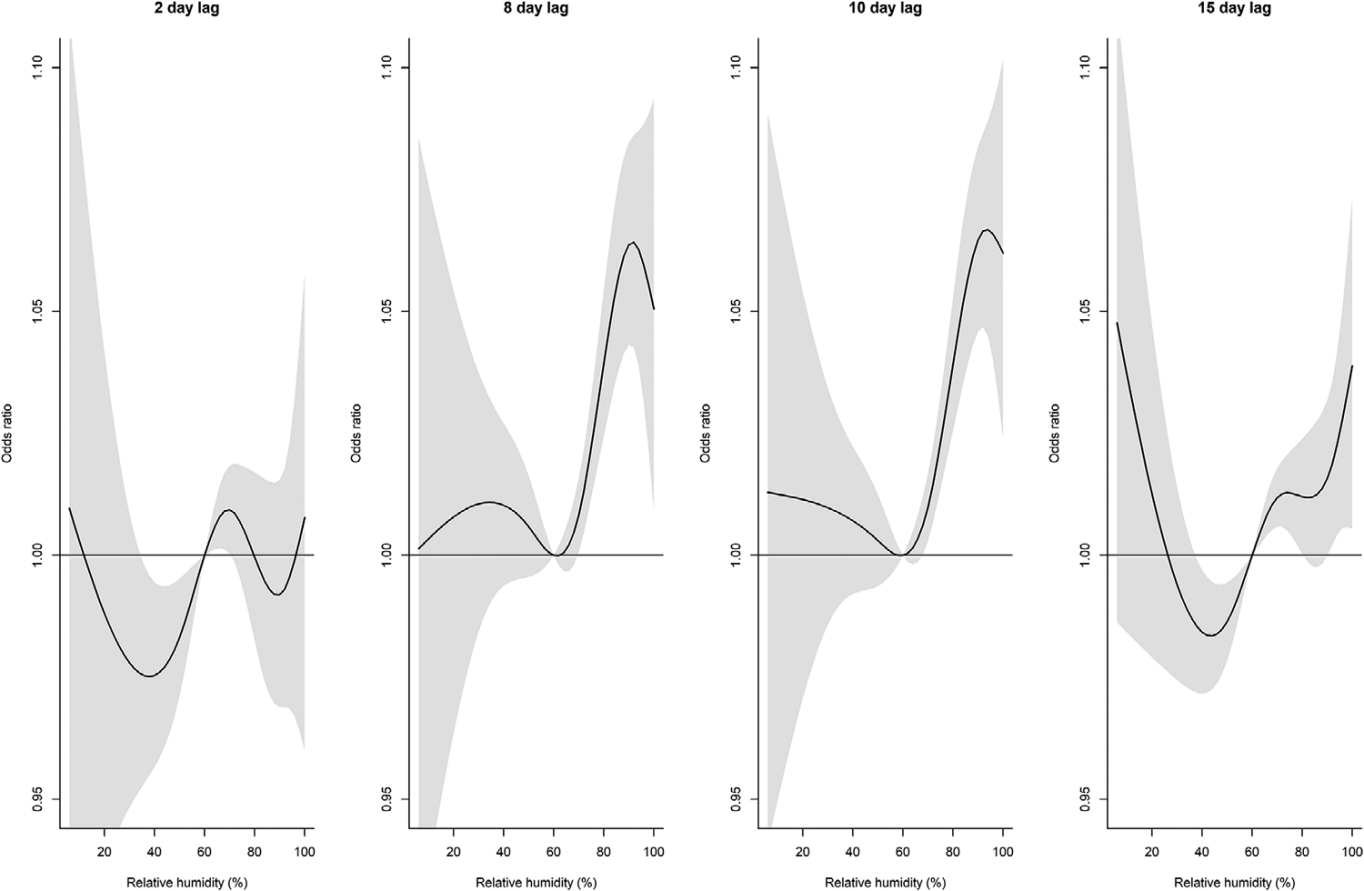
Distributed lag non-linear model for legionellosis cases. Effect of relative humidity at selected lag days (odds ratios are relative to 60% relative humidity).

**Figure 10.**
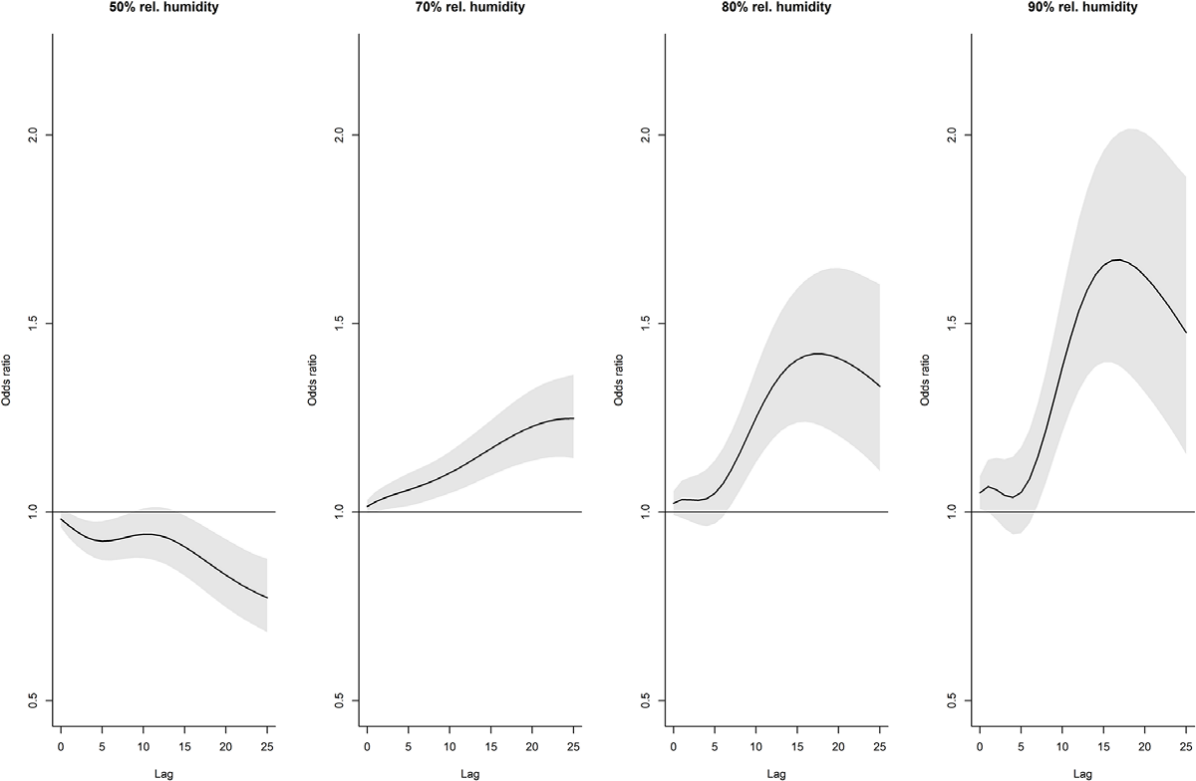
Distributed lag non-linear model for legionellosis cases. Effect of selected relative humidity levels over the lag period (odds ratios relative to 60% relative humidity).

At lag days 8 and 10, compared to 60% relative humidity, odds ratios increased from 60% to above 90% but declined thereafter (Figure 9). No obvious patterns were seen at lag days 2 and 15. For relative humidity of 80% and 90%, there was an increased effect on lag days 6–15, declining thereafter, with stronger effects evident at 90% (Figure 10). The cumulative effect of 90% relative humidity was a 1.7 times increased odds of legionellosis (95% CI 1.39–2.01) over 17 days (Supplementary Figure S10).

**Figure 11.**
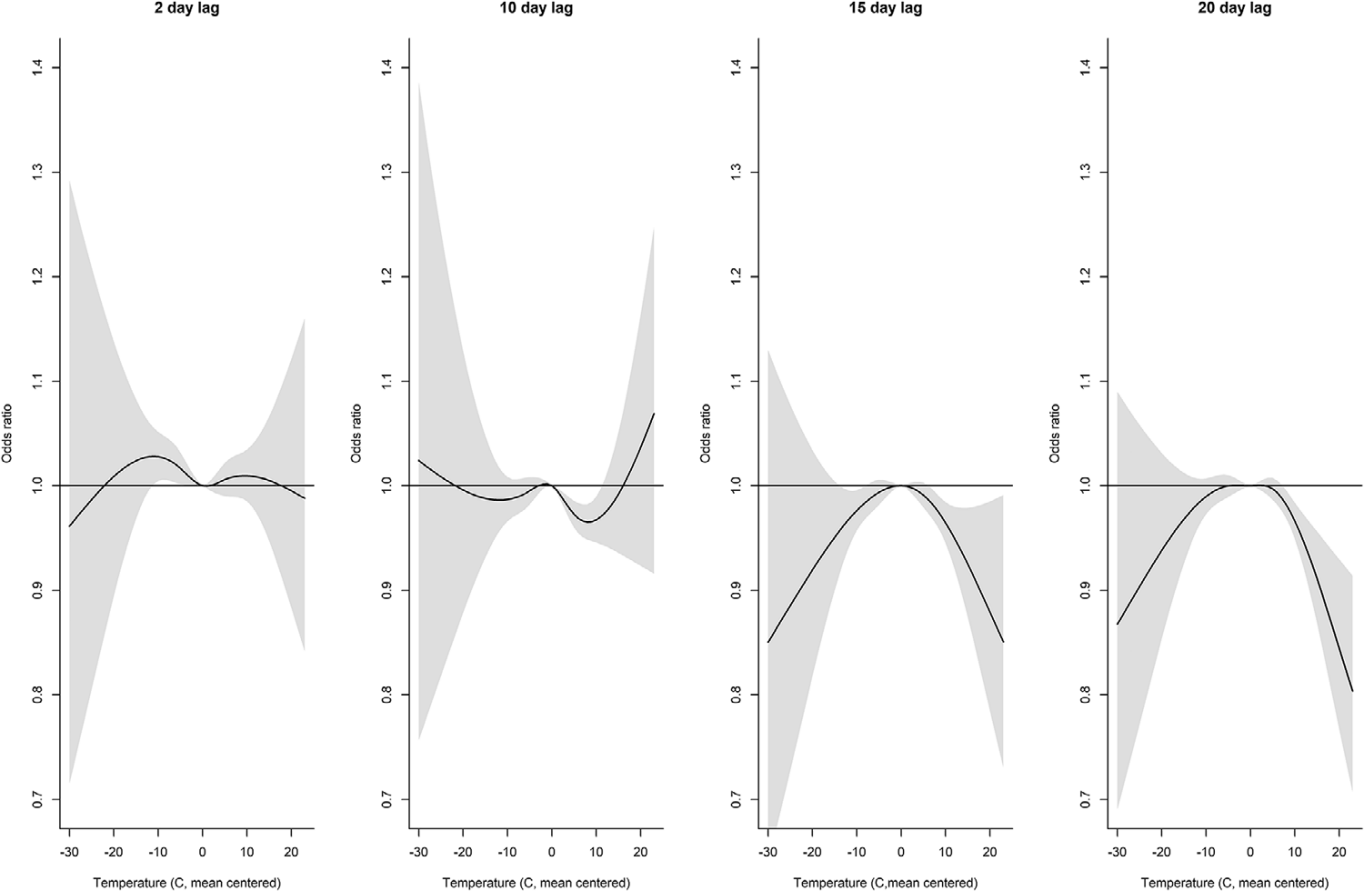
Distributed lag non-linear model for legionellosis cases. Effect of daily temperature (°C, mean centred) at selected lag days (odds ratios are relative to mean temperature).

**Figure 12.**
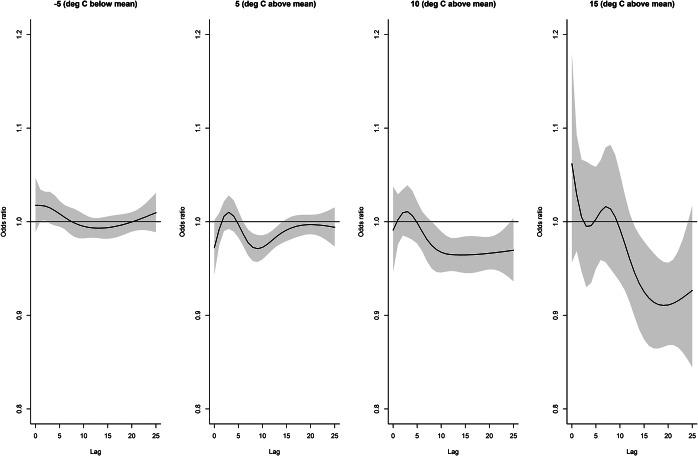
Distributed lag non-linear model for legionellosis cases. Effects at selected daily maximum (mean-centred) temperatures (°C) over lag period (odds ratios relative to mean temperature).

For mean-centred temperatures at lags of 15 and 20 days, there was some evidence of a slight inverse association starting at 6 to 8°C above the mean (Figure 11). Figure 12 also illustrates a slight inverse association at mean temperatures 5°C above the mean for lags 6–15; at 10°C above the mean starting at lag day 9; and at 15°C above the mean starting at lag day 14. The cumulative effect of 10°C above the mean halved the odds of legionellosis over a 20–25 day lag period (Supplementary Figure S11).

For the threshold models of high days, days of precipitation above 1.27 cm (0.5 inches) and humidity >90%, were associated with increased legionellosis from lag days 5–20, peaking from lag days 11–18 (Supplementary Figure S12). The cumulative effect of a day of precipitation above 1.27 cm increased the odds of legionellosis over five times over a 20 day period (not shown), whereas a day of humidity above 90% was associated with a 1.7 increase in the odds of legionellosis over 20 days (not shown). High temperature showed decreasing risks occurring a few days prior to the increased risks from humidity and temperature, beginning at lag day 10 (Supplementary Figure S12).

## Discussion

Precipitation and humidity at lags of 8–13 days were associated with an increased risk of hospitalization for legionellosis among those receiving Medicare. The association with precipitation showed a clear peak for lag days 8 through 13, extending to 17 days for days of precipitation greater than 2.54 cm or 1 inch. The effect of humidity was more apparent for the threshold effect of high days, with significant associations at lag days 9 and 11 for days of humidity greater than 80%. These lags are consistent with the expected duration between exposure to the *Legionella*, incubation, and reporting to a hospital. However, the association varied by geography and season. The association was absent in the western and southwestern states, and most pronounced in the northeastern and midwestern states. Associations were strongest in the Fall and Summer and generally absent in the Spring and Winter. Both observations reflect where and when most legionellosis cases occur.

Distributed lag non-linear models revealed additional insights. The effect of precipitation peaked and then declined above 7 cm. This amount of precipitation is likely associated with major storms possibly with high winds and storm surges that may not be favourable to the transmission and survival of *Legionella.* However, there were too few observations to make firm conclusions. Humidity also appeared to show a threshold peaking at 93%–94%. There was some reduced risk associated with increasing temperatures. This could be due to inverse correlations between temperature and humidity and precipitation that were not fully controlled. The inverse effect of temperature appeared to precede the effect of precipitation (beginning about lag day 15). One interpretation may be that cooler temperatures around lag day 15, followed by precipitation and humidity at lag days 8–13 was a pattern that increased legionellosis risk.

As discussed previously [18], DLNMs can be sensitive to the distributions used for the lag and response, and we did not do a comprehensive comparison of different distributions. Using an alternate lag structure with knots at 5, 10, and 15 days did not affect the results appreciably. For example, the 20-day cumulative effect of 3 cm of precipitation increased the odds of legionellosis was nearly identical (OR = 4.5, 95% CI 2.98–6.71) to the 20-day cumulative effect of the original model with knots at 2, 7, and 14 days (OR = 4.4, 95% CI 2.94–6.60). The peaks and the shape of the association also remained the same, however the slight inverse association on lag days 1–3 was attenuated (Supplementary Figure S13). The cumulative effects of relative humidity and temperature were also not significantly affected under a model with knots at 5, 10, and 15 days (not shown) although some variation in the associations are seen at the locations of the knots (Supplementary Figures S14 and S15). Different lag distributions and response functions could affect the findings, however, for this exploratory analysis, we selected knots *a priori* based on the known incubation period of *Legionella* infection and at percentiles of the exposure variables. Because of the complex relationship between legionellosis hospitalization and weather condition, the true lag structure is not known, and there are too many potential possibilities to fully explore in this analysis.

The study supports the hypothesis that wet and humid conditions favour the survival of *Legionella* in the environment and increase the likelihood of ambient human exposure and subsequent infection. Although local weather conditions probably cannot explain outbreaks in hospitals, nursing homes, hotels, and so on, they may contribute to the much larger proportion of sporadic cases. The wet and humid conditions that favour *Legionella* survival also may explain the geographic patterns we observed as associations were absent in the dryer West and Southwest regions.

Some other studies have observed positive associations with temperature, but others have observed inverse, negative or no associations between Legionnaires’ Disease and temperature [25]. Legionnaires’ Disease demonstrates a distinct seasonality and temperature is strongly correlated with season, making it difficult to disentangle their independent effects.

This is one of the largest studies of legionellosis and weather, representing the entire Medicare eligible population for a time-period of 20 years across the continental United States. We used a conservative statistical approach by using the Bonferroni method to adjust *p*-values and confidence intervals. Although this approach reduced the likelihood of Type I errors, it may have overlooked some true effects. For example, some effects of relative humidity were significant without Bonferroni correction but after correction failed to meet significance criteria.

Because of the expansive geography and time-period of this study, we were unable to obtain factors such as wind speed and direction and air quality measures. Few other studies have included these factors and those that have observed mixed associations [25]. The case-crossover approach controls for medium and longer-term trends in air quality and wind as we matched controls within calendar year and month (within two weeks), although day to day variations would not be accounted for completely.

Although we took measures to address collinearity we may not have been able to fully account for correlated predictors in the additive models. Correlation among predictor variables would not bias effect estimates but would reduce their precision. There was some evidence of this, because single-exposure models for relative humidity showed more significant associations than multiple-exposure models.

Legionellosis hospitalizations among the Medicare population are an underestimate true cases of legionellosis, especially Pontiac Fever. There were very few cases of Pontiac Fever without a corresponding diagnosis for Legionnaires’ Diseases, so our findings should not be extrapolated to Pontiac Fever cases alone. Legionnaires’ Disease is often severe in those over 65 and many likely require hospitalization. Legionellosis is a nationally notifiable disease in the United States and according to the CDC, in 2018 there were 2918 cases over 70 years of age (A [37]). We identified 2303 cases over 70 years of age in 2018, representing 80% of the cases identified by CDC. Although the use of hospitalization ICD codes may result in misclassification, this was unlikely to systematically bias our findings.

Our study linked daily weather to cases based on zip code level geography, which is an improvement over studies which aggregated weather and/or cases by week, month, county, or state. This allowed for detailed modelling of lags, including assessments of non-linear trends in the exposure and lags. The weather data was based on the best available source. Estimates from the PRISM group at Oregon State University are well-documented and widely used in climate research.

In conclusion, we found that precipitation and relative humidity at lags between 8 through 13 days were associated with an increased risk of hospitalization for legionellosis among the Medicare-eligible population in the United States representing nearly 40 000 cases over a 20 year period from 2000 through 2020. This lag is consistent with time frame that would be expected from exposure, infection, incubation to fulminant disease and resulting hospitalization resulting from *Legionella.*

## Supporting information

Wade and Herbert supplementary material Wade and Herbert supplementary material

## Data Availability

The data that support the findings of this study are available from the Centers for Medicare and Medicaid Services (CMS). Restrictions apply to the availability of these data, which were provided under a Data Use Agreement specific to this study. Data are available from: https://www.cms.gov/data-research/files-for-order/data-disclosures-and-data-use-agreements-duas/limited-data-set-lds with the requirement of a signed Data Use Agreement. Data do not contain personally identifiable information but contain are classified as Limited Data Set files and their distribution requires an agreement and between CMS and the requester and approval by CMS. Weather data are available at https://prism.oregonstate.edu/. Because the data do not contain identifiable private information and were not obtained through interaction or intervention with individuals, the Institutional Review Board for the University of North Carolina and the US Environmental Protection Agency Human Research Protocol Officer determined that the use of this data does not constitute human subjects research.

## Supplementary material

For supplementary material accompanying this paper visit http://doi.org/10.1017/S0950268824000979. click here to view supplementary material
