# Supplementary material for: Weather conditions and legionellosis: a nationwide case-crossover study among Medicare recipients
Source: Epidemiol Infect. 2024 Oct 17;152:e125. doi: 10.1017/S0950268824000979 (PMC11502464; doi:10.1017/S0950268824000979)
Supplement: Wade and Herbert supplementary material [file S0950268824000979sup001.docx]

Supplementary Material

Epidemiology and Infection

Weather conditions and Legionellosis: A nationwide case-crossover study among Medicare recipients

Timothy J. Wade and Carly Herbert

Figure S1: Legionellosis hospitalizations among Medicare recipients, age adjusted rates per 100,000 (2010-2020).

Figure S2: Associations between Legionellosis cases and daily weather from conditional logistic regression case-crossover model, single exposure models. Odds ratios for relative humidity are for 5% increase; precipitation for a 1 cm increase, and temperature for 35 ^o^C increase.

Figure S3: Associations between Legionellosis cases and high weather days from conditional logistic regression case-crossover model, single exposure models. Type 1: any precipitation; relative humidity>80% and maximum daily temperature > 29 ^o^C; Type 2: daily precipitation>1.27 cm (0.5 inch); relative humidity>90% and maximum daily temperature > 32 ^o^C; and Type 3: precipitation>2.54 cm (1 inch) relative humidity>95% and maximum daily temperature > 35 ^o^C.

Figure S4: Associations between Legionellosis cases and temperature (odds ratios per 3 ^o^C increase) from conditional logistic regression case-crossover model, by region.

Figure S5: Associations between Legionellosis cases and high weather days from conditional logistic regression case-crossover model, by region. High days (definition 3): precipitation>2.54 cm (1 inch); relative humidity>95% and maximum daily temperature > 35 ^o^C.

Figure S6: Associations between Legionellosis cases and precipitation and humidity, from conditional logistic regression case-crossover model, by season. Odds ratios for relative humidity are for 5% increase; precipitation for a 1 cm increase, and temperature for 3 ^o^C increase.

Figure S7: Associations between Legionellosis cases and high weather days from conditional logistic regression case-crossover model, by season. Odds ratios are for days of precipitation>1.27 cm (0.5 inch) and relative humidity>90%.

Figure S8: Associations between Legionellosis cases and weather, from case-crossover model, by year.  Odds ratios for relative humidity are for 5% increase; precipitation for a 1 cm increase, and temperature for 3 ^o^C increase.

Figure S9: Distributed lag non-linear model for Legionellosis cases. Cumulative effect of daily precipitation (odds ratios are relative to no precipitation).

**

Figure S10: Distributed lag non-linear model for Legionellosis cases. Cumulative effect of relative humidity (odds ratios are relative to 60% relative humidity).

**

Figure S11: Distributed lag non-linear model for Legionellosis cases. Cumulative effect of temperature (odds ratios are relative to mean temperature).

**

Figure S12: Distributed lag model for legionellosis cases. Days of high precipitation >1.27 cm (0.5 inch); relative humidity>90%; and temperature >32 ^o^C.

**

Figure S13: Distributed lag model for legionellosis cases. Effect of daily precipitation for selected totals over the lag period, with lag spline distribution knots at 5, 10 and 15 days (odds ratios relative to no precipitation).

Figure S14: Distributed lag model for legionellosis cases. Effect at selected humidity levels over the lag period, with lag spline distribution knots at 5, 10 and 15 days (odds ratios relative to 60% humidity).

Figure S15: Distributed lag model for legionellosis cases. Effects at selected daily maximum temperatures (mean-centered) levels over the lag period, with lag spline distribution knots at 5, 10 and 15 days (odds ratios are relative to mean-centered temperature).

**
